# Breast Augmentation

**Published:** 2013-06-13

**Authors:** Sachin M. Shridharani, Justin L. Bellamy, Mark M. Mofid, Navin K. Singh

**Affiliations:** Department of Plastic Surgery, The Johns Hopkins University School of Medicine, Baltimore, Md

**Figure:**
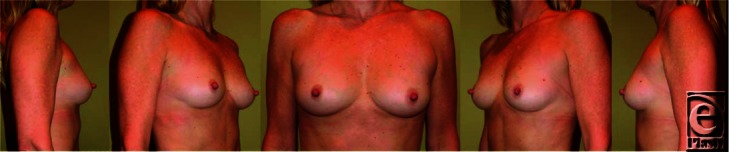


## DESCRIPTION

A 28-year-old woman presents to the plastic surgeon's office for consultation regarding her desire for larger breast. She is overall healthy, with a nonsignificant medical history. Her height and weight are 5′8″ and 60 kg, respectively.

## QUESTIONS

1. **What are the available options for breast implants? What are their advantages and disadvantages?**
2. **What are the common surgical approaches for breast augmentation? What are their advantages and disadvantages?**
3. **What is capsular contracture?**
4. **How does one diagnose an implant rupture?**
5. **Does breast augmentation surgery impact breast cancer screening with mammography?**

## DISCUSSION

Since 2006, augmentation mammoplasty has remained the most commonly performed cosmetic surgical procedure in the United States, with nearly 300 000 patients annually undergoing the procedure.^1^ The modern era of silicone elastomer-shelled prostheses (breast implants filled with either silicone or saline) began in early 1960s and has seen fluctuation in practice trends regarding which implant to use. A thorough understanding of the most recent recommendations remains important in providing the best short- and long-term care for patients desiring breast augmentation.

The 2 primary types of breast implants used today are saline- or silicone-filled. In 1992, the US Food and Drug Administration issued a moratorium on silicone implants because of an alleged link to autoimmune disease. Surgeons defaulted to the use of saline-filled implants as the predominant implant; however, these implants have otherwise been considered a second choice to silicone implants in the United States and abroad because of increased rupture/deflation rates and problems with underfilling and overfilling. These issues may result in a need for revision surgery and dissatisfactory aesthetic result. After extensive population-based studies failed to show a correlation between autoimmune disease and silicone implants, the moratorium was lifted in 2006, and silicone breast implants came back into popularity in the United States.^2^ The most recent generation of silicone implants features a barrier layer to reduce the silicone bleeding phenomenon seen in older implants. Textured implants were initially developed to mimic polyurethane implant shells that were shown to have very low rates of capsular contracture but are not available in the United States. Unfortunately, there is little high-level evidence that surface texturizing alone reduces capsular contracture.^3^^,^^4^ The primary utility of texturized implants is to reduce implant rotation in anatomic-shaped prostheses. Innovation regarding silicone chemical processing, including extensive cross-linking of the filler, has led to the development of cohesive gel implants. Cohesive gel implants are supple, yet able to maintain their shape without being deformed by the surrounding soft-tissue envelope or gravitational effects over time. In addition to aesthetic benefit, these “form stable” or “gummy-bear” implants may reduce long-term implant-related complications.^5^^-^7

Implants are available in 2 general shapes: round or anatomic. The indications for either type vary, and surgeons should be comfortable using one or the other as dictated by patient goals and anatomy. Round implants may be suitable for patients when rotation of an anatomic-shaped implant would be of concern. These circumstances include patients exhibiting increased athletic activity and revision surgery after confirmed rotation deformity. In addition, patients deemed candidates suited for round implants include those patients in whom the round shape would be less noticeable (fuller patients, patients with good skin quality, or patients requiring smaller volumes). Furthermore, patients who explicitly desire an overfilled or large breast appearance may be excellent candidates for round implants. Anatomic-shaped implants are well suited for patients desiring a natural appearance or those with mild ptosis or pseudoptosis. One should note, however, that a “natural” appearance can be achieved with either shape, and significant ptosis with increased skin envelope laxity may lead to increased risk of rotation with anatomic implants. Patients with constricted lower-pole breasts and thoracic hypoplasia may also benefit from form-stable anatomic-shaped implants. Many surgeons have advocated for tissue-based planning—preoperative breast dimensional measurements dictating implant selection. These efforts have improved outcomes and reduced reoperation. The goal of tissue-based planning is to select an implant that will fill the breast while simultaneously respecting natural anatomy, matching the breast footplate, and minimizing breast distortion.^8^^-^10 Rather than cup-size, the width and skin-stretch measurements of the soft tissue envelope are used to determine optimal fill volume.^9^ Alternatively, a saline-filled breast sizer can be inserted, filled, and removed intraoperatively to objectively determine the optimal volume for the permanent implant.

Several access incisions/approaches are employed: inframammary, periareolar, transaxillary, and transumbilical (saline only). There are advantages and disadvantages to each approach. Inframammary incisions provide the most control, allowing the surgeon to set the location of the inframammary fold while minimizing implant trauma or contamination; however, without careful planning the scar may not be ideal. The periareolar incision allows good access and disguise of the scar; however, the incision may increase implant contamination (due to transection of parenchymal ducts often colonized by *Staphylococcus epidermidis*)^11^ and resultant capsular contracture. Further, the scar is placed in the central area of interest in the breast, and for those prone to hypertrophic scarring, this would be a riskier incisional choice. The transaxillary approach results in no scar on the breast; however, the remote access site reduces control for pectoralis major release unless endoscopically assisted. Furthermore, placing larger silicone gel implants can be challenging via the transaxillary approach because of limited incision size. Should revision surgery be required after transaxillary or transumbilical approaches, inframammary incisions are often ultimately required.

The main surgical planes of implant placement are subglandular or subpectoral/submuscular with release of the inferior pectoral origin. Total submuscular planes of dissection have been described but have limited indications. Subglandular placement of implants may be appropriate in patients with sufficient overlying soft tissue in the upper pole of the breast (determined by a pinch test of >1-2 cm). The subglandular space is viewed by some to represent a more natural plane yielding a more natural-appearing augmentation.^12^ In addition, placing implants in this plane allows for correction of mild breast ptosis without having to perform a separate procedure on the breast mound to elevate the nipple-areola complex. Alternatively, subpectoral implant placement provides its own benefits. There is some evidence to suggest that it may provide improved capsular contracture rates,^13^ although high-level evidence is limited. Similarly, it has been suggested that subpectoral placement may improve breast visibility on mammography.^14^ In recent years, the subpectoral approach has been refined, using the dual plane technique to combine good upper and medial cover with improved draping of the lower pole over the implant and less pectoralis animation.^15^ Three types of dual plane approaches have been described, with each level describing increasing degree of release of anterior pectoral fascial attachments from overlying glandular tissue. Dual plane I features division of the inferior pectoral origin without further fascial release, dual plane II adds release of anterior pectoral fascial attachments to the level of the inferior areolar border and rotation of the inferior origin of the pectoralis, while dual plane III involves fascial release and rotation at the level of the superior areolar border.

Regardless of the surgical approach, the surgeon should be familiar with complications specific to breast augmentation. Capsular contracture remains one of the most troubling and frequently reported complications of augmentation mammoplasty, and thus reduction in its occurrence has been the topic of numerous studies.^16^ Breast prostheses, being a foreign body, invariably develop a capsule as a protective immune reaction. Capsular contracture describes the pathologic activation of this capsule that results in a constrictive fibrosis that deforms and impairs the aesthetic result. Several causal theories, involving endogenous (eg, due to host-implant interaction) and exogenous (eg, due to subclinical infection, biofilms) contributions, have been proposed, although the exact cause is multifactorial and remains elusive. Baker's original clinical classification of capsular contracture remains a simple tool to describe severity, where grades I (normal capsule) and II (palpable but nonvisible) are considered acceptable and grades III (palpable and visible) and IV (painful, hard, and very constrictive) represent pathologic contracture.^17^ While capsular contracture mostly happens within the first 2 years, there is a long-term cumulative increase in capsular contracture risk over time^18^^-^20 that may require surgical capsulectomy with implant replacement.

An important consideration after augmentation mammoplasty includes breast imaging. First, the surgeon should be aware of the possibility of implant rupture. Intracapsular implant rupture (those not extending beyond the fibrous capsule around the implant) represents 80% to 90% of implant ruptures.^21^^,^^22^ Diagnosis is generally more straightforward with saline-filled implants. Rupture is followed quickly by total/near-total deflation. If patients have silicone-filled breast implants, these ruptures can be diagnosed with magnetic resonance imaging by the presence of multiple curvilinear low-signal-intensity lines within the silicone gel (known as “linguine sign”) on T2-weighted imaging. The rarer extracapsular rupture can be identified by identification of free, high-signal-intensity silicone in surrounding breast tissue. Second, imaging considerations for breast cancer screening remains an important issue after augmentation. While many studies suggest a trend toward lower cancer rates in patients with implants (perhaps reflecting the patient population, smaller breast size, etc), breast prostheses do create certain mammography challenges. Both silicone and saline implants create dense radio-opaque shadows and limit maximal compression of breast tissue during standard mammography that may interfere with detection of smaller subtle lesions.^23^ Capsular contracture can further reduce sensitivity by as much as 30% with Baker I/II, or by more than 50% with Baker III/IV.^14^^,^^24^ To counteract these effects, the Eklund Pushback technique,^25^ which involves pushing the implant posteriorly toward the chest wall and pulling the breast tissue forward, can be employed by the radiologist to significantly increase the amount of breast tissue visualized. Despite this theoretical hindrance to optimal mammography screening, studies have failed to demonstrate any significant delay in cancer detection.^26^ In addition, overall breast cancer survival rates are ultimately not impacted by breast augmentation.^27^ Last, the increasing use of magnetic resonance imaging in breast imaging may obviate the limitations associated with mammography.

**Figure:**
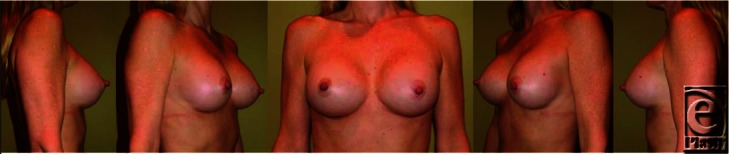


## DESCRIPTION

The patient underwent placement of 300 mL round, smooth, silicone gel–filled implants through an inframammary fold incision in a dual plane approach. She did well in the postoperative picture and was pleased with the overall aesthetic outcome. Given earlier is her picture at 1-year follow-up.
